# Fluorescence detection of DNA mismatch repair in human cells

**DOI:** 10.1038/s41598-018-30733-x

**Published:** 2018-08-15

**Authors:** Shunsuke Ito, Miyako Shiraishi, Kazuki Tsuchihashi, Reine Takatsuka, Junpei Yamamoto, Isao Kuraoka, Shigenori Iwai

**Affiliations:** 1 Division of Chemistry, Graduate School of Engineering Science, Osaka University, 1-3 Machikaneyama, Toyonaka, Osaka, 560-8531 Japan; 2 Present Address: Department of Chemistry, Faculty of Science, Fukuoka University, 8-19-1 Nanakuma, Jonan-ku, Fukuoka, 814-0180 Japan

## Abstract

Mismatched base pairs, produced by nucleotide misincorporation by DNA polymerase, are repaired by the mismatch repair (MMR) pathway to maintain genetic integrity. We have developed a method for the fluorescence detection of the cellular MMR ability. A mismatch, which would generate a stop codon in the mRNA transcript unless it was repaired, was introduced into the gene encoding the enhanced green fluorescent protein (EGFP) in an expression plasmid. When MMR-proficient HeLa cells were transformed with this plasmid, the production of active EGFP was observed by fluorescence microscopy. It was assumed that the nick required to initiate the MMR pathway was produced non-specifically in the cells. In contrast, fluorescence was not detected for three types of MMR-deficient cells, LoVo, HCT116, and DLD-1, transformed with the same plasmid. In addition, the expression of a red fluorescent protein gene was utilized to avoid false-negative results. This simple fluorescence method may improve the detection of repair defects, as a biomarker for cancer diagnosis and therapy.

## Introduction

DNA must be replicated prior to cell division. In eukaryotic cells, three DNA polymerases, α, δ, and ε, are primarily involved in the replication^1^. DNA polymerase α initiates the replication with its primase activity, and DNA polymerases δ and ε synthesize the lagging and leading strands, respectively, by nucleotide polymerization in the 5′ to 3′ direction, depending on the base-pair formation with the template strand. This process must be accurate because the misincorporation of a nucleotide, to form a mismatch, would induce a genetic mutation, which results in carcinogenesis or cell death. Although DNA polymerases δ and ε have proofreading activities that remove the wrong nucleotide and reincorporate a nucleotide, they still produce one mismatch per about 10^6^ nucleotides^2^.

The mismatched base pairs are repaired by the mismatch repair (MMR) pathway^3–5^. In prokaryotic cells, the dimeric MutS protein recognizes the mismatch by clamping and kinking the DNA with its two monomers^6^. MutL is recruited to the MutS–DNA complex, and the MutS–MutL complex moves along the DNA and activates the MutH endonuclease. At the mismatch site, both of the bases in the two strands are normal, unlike the damaged bases induced by ultraviolet light, oxidative stress, and so on, and the nucleotide in the newly-synthesized strand must be removed. MutH recognizes the *N*^6^-methyadenine in the GATC sequence, produced by DNA adenine methyltransferase, to identify the template strand in the replication, and cleaves the unmethylated strand on either the 5′ or 3′ side of the mismatch. From this incision site, DNA helicase II, encoded by the *uvrD* gene, unwinds the DNA, and four single-strand nucleases, RecJ, ExoI, ExoVII, and ExoX, digest the cleaved strand. Finally, the single-stranded region is filled in by DNA polymerase III, and the gap is sealed by DNA ligase. In eukaryotes, MutSα, a heterodimer of two MutS homologs (MSH2 and MSH6), recognizes the mismatch in a similar manner to the prokaryotic MutS^7^. Another important factor that functions in eukaryotic MMR is MutLα, a heterodimer of the MutL homolog 1 (MLH1) and postmeiotic segregation increased 2 (PMS2) proteins, which binds to MutSα. MutSα moves along the DNA in an ATP-dependent manner, and MutLα has endonuclease activity that is activated by proliferating cell nuclear antigen (PCNA) binding, to cleave the DNA strand containing an initial nick^8^. Regardless of the 5′ and 3′ locations of the initial nick, the PCNA-bound MutSα–MutLα complex makes incisions on both the 5′ and 3′ sides of the mismatch^9^. The terminus of the Okazaki fragment may be the initial nick required for the strand discrimination^10^, and another possibility is the ribonuclease H2-mediated cleavage at ribonucleotides misincorporated into DNA^11,12^. From the incision on the 5′ side of the mismatch, ExoI degrades the strand in the 3′ direction past the mismatch site, and then repair synthesis by DNA polymerase δ occurs. Even in the absence of ExoI, the strand displacement by DNA polymerase δ can complete MMR^13^.

Defects in DNA repair pathways cause diseases, and in the case of MMR, Lynch syndrome^14,15^, which was previously referred to as hereditary non-polyposis colorectal cancer, is a typical example. It is the most common inherited colorectal cancer syndrome, and is caused by germline mutations in one of the four MMR genes, *MLH1*, *MSH2*, *MSH6*, and *PMS2*. A somatic mutation of the wild-type allele results in the loss of the MMR function, and leads to colorectal cancer, as well as endometrial cancer. Since Lynch syndrome is an autosomal dominant disorder, its diagnosis is important, and surveillance is required for family members of patients diagnosed with this syndrome. To detect the MMR deficiency, the microsatellite instability (MSI) is analyzed^16,17^. The alterations in the lengths of simple repeat sequences, called microsatellites, correlate with the MMR function, and according to the ratios of the length alterations found in the analyzed microsatellites, the results are classified into three categories, MSI-H (MSI-high), MSI-L (MSI-low), and MSS (microsatellite stable)^18^. The MSI-H phenotype strongly supports the Lynch syndrome diagnosis. An alternative method for the screening of Lynch syndrome is the analysis of the MMR proteins by immunohistochemistry (IHC). However, neither of these tests can definitively detect the MMR deficiency^17^. For example, the majority of tumors from *MSH6*-mutation carriers do not have MSI-H^19^, and the complex relationship between MMR defects and MSI patterns has also been noted^20^. In IHC, false-positive results may be obtained for dysfunctional MMR gene products containing a single amino acid substitution, particularly outside the dimerization domains^21^.

**Figure 1:**
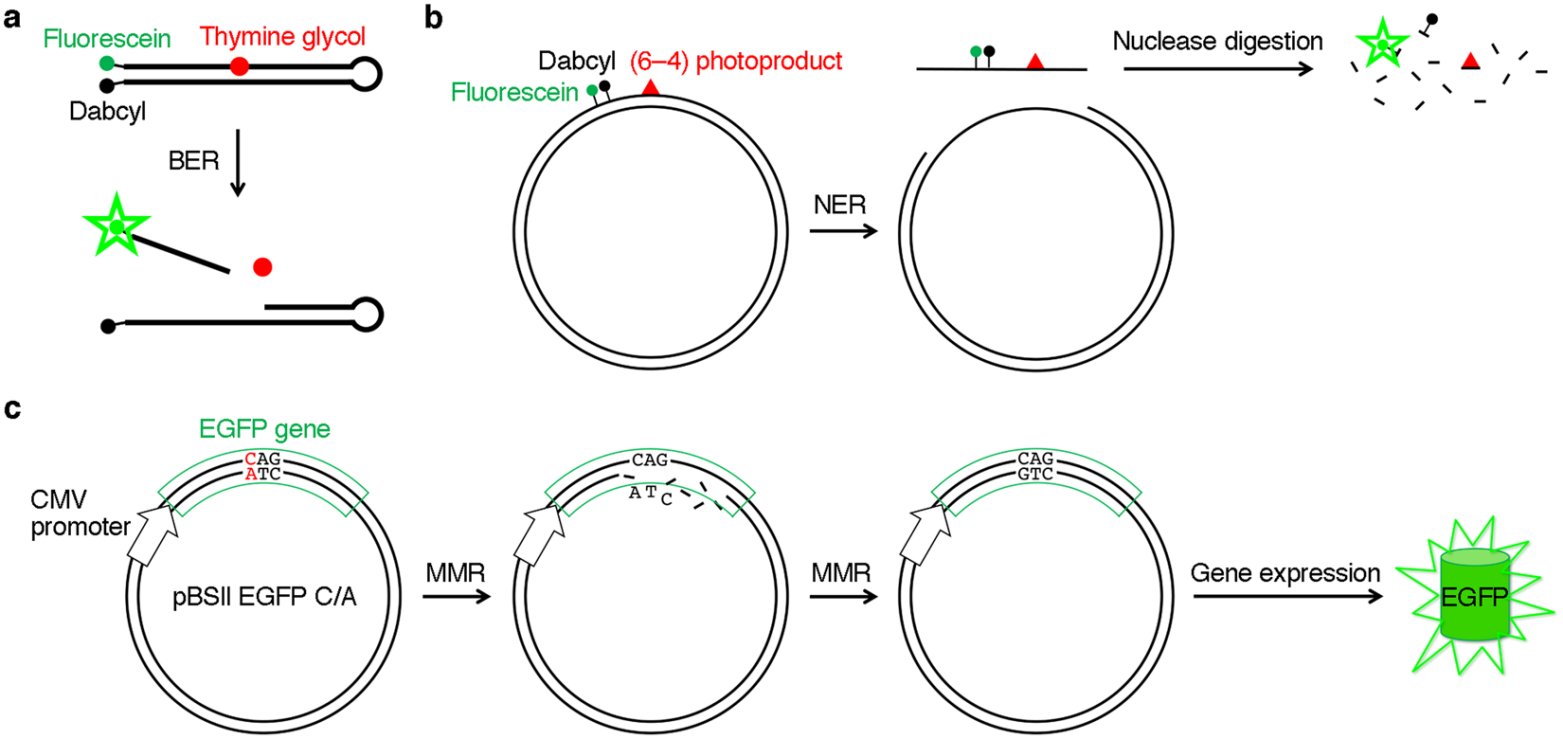
Fluorescence detection of DNA repair in cells. (**a**) Detection of base excision repair developed in our previous study^22^. The probe contained an oxidatively damaged base in the center and a fluorophore–quencher pair at the end, and the phosphodiester linkages were changed to nuclease-resistant phosphorothioate analogs in the regions that were not required for enzyme binding. Fluorescence was detected when the probe was cleaved by NTH1 in human cells. (**b**) Detection of nucleotide excision repair developed in our previous study^23^. A plasmid was used as a scaffold, and the fluorophore and the quencher were attached to the base moieties near a UV-induced lesion. Fluorescence was detected when the dual-incision product was degraded in cells. (**c**) Detection of MMR in this study. Cells are transformed with a plasmid containing a mismatch (shown in red) in the EGFP gene, which is expressed under the control of the CMV promoter. In the absence of MMR, the mRNA transcript contains a stop codon, UAG. However, when the cells are MMR-proficient, the repair of the bottom strand results in the generation of the original EGFP gene, and fluorescence is detected in the cells. We expected that the initial nick required for MMR would be produced in the cells in a random manner. There is a possibility that the top strand is repaired, but the full-length EGFP is not produced in this case.

In this study, we developed a method to detect the cellular MMR ability by fluorescence. We previously reported the fluorescence-mediated detection of the base excision repair (BER)^22^ and nucleotide excision repair (NER)^23^ of DNA lesions induced by oxidative stress and UV irradiation, respectively, in cultured human cells (Fig. 1a,b). In both cases, a fluorophore–quencher pair, which was originally used in a molecular beacon^24^, was incorporated into a hairpin-shaped oligonucleotide or a plasmid containing a damaged base. In the present study, the expression of the gene encoding green fluorescent protein, containing a mismatch, was utilized to detect the overall process of MMR (Fig. 1c).

## Results

### Preparation of the expression plasmid containing a mismatch

In this study, we intended to develop a system in which a mismatch-containing gene encoding a full-length fluorescent protein is expressed in cells only when it is repaired, to detect the cellular MMR ability. We constructed a plasmid (pBSII EGFP) to express the gene encoding the enhanced green fluorescent protein (EGFP), which has the F64L and S65T substitutions in the original green fluorescent protein^25^, and then introduced a C/A mismatch at the first position of the codon for Q157 (CAG) of this protein (pBSII EGFP C/A, Fig. 1c). The T/G mismatch was avoided because it could be recognized by other enzymes, such as the methyl-CpG domain protein and thymine DNA glycosylase^26^, and the C/A mismatch is a good substrate for the MMR pathway^27^. Since the template strand had the sequence 3′-ATC-5′, the mRNA produced under the control of the cytomegalovirus (CMV) promoter in the cells transformed with this plasmid would contain a stop codon, UAG, in the absence of the cellular MMR activity. In MMR-proficient cells, we expected that two types of plasmids would be generated. One of them should contain the C·G pair at the first position of this codon, produced by the removal of the A in the bottom strand to be used as the template in transcription, as shown in Fig. 1c. Since the CAG sequence at this site in the mRNA produced from the repaired plasmid is the codon for glutamine, the full-length EGFP would be produced. In other words, fluorescence would be observed, depending on the cellular MMR ability. An expression plasmid containing the normal EGFP gene without the mismatch (pBSII EGFP C·G) was also prepared in the same manner.

In order to initiate the MMR pathway, not only a mismatch but also a nick is required in the DNA, as described in the Introduction. At the beginning of this study, we planned to either incorporate a ribonucleotide^11,12^ into the mismatch-containing primer used for the preparation of the double-stranded plasmid from the single-stranded DNA, or introduce a nick into the plasmid with a nicking endonuclease^28^. However, we previously found that the plasmids were degraded in the cells within several hours during the culture at 37 °C^23^. Therefore, we decided to test the plasmids without the nick, and expected that a nick would be produced non-specifically in the cells.

### Transformation of MMR-proficient and MMR-deficient cells

**Figure 2:**
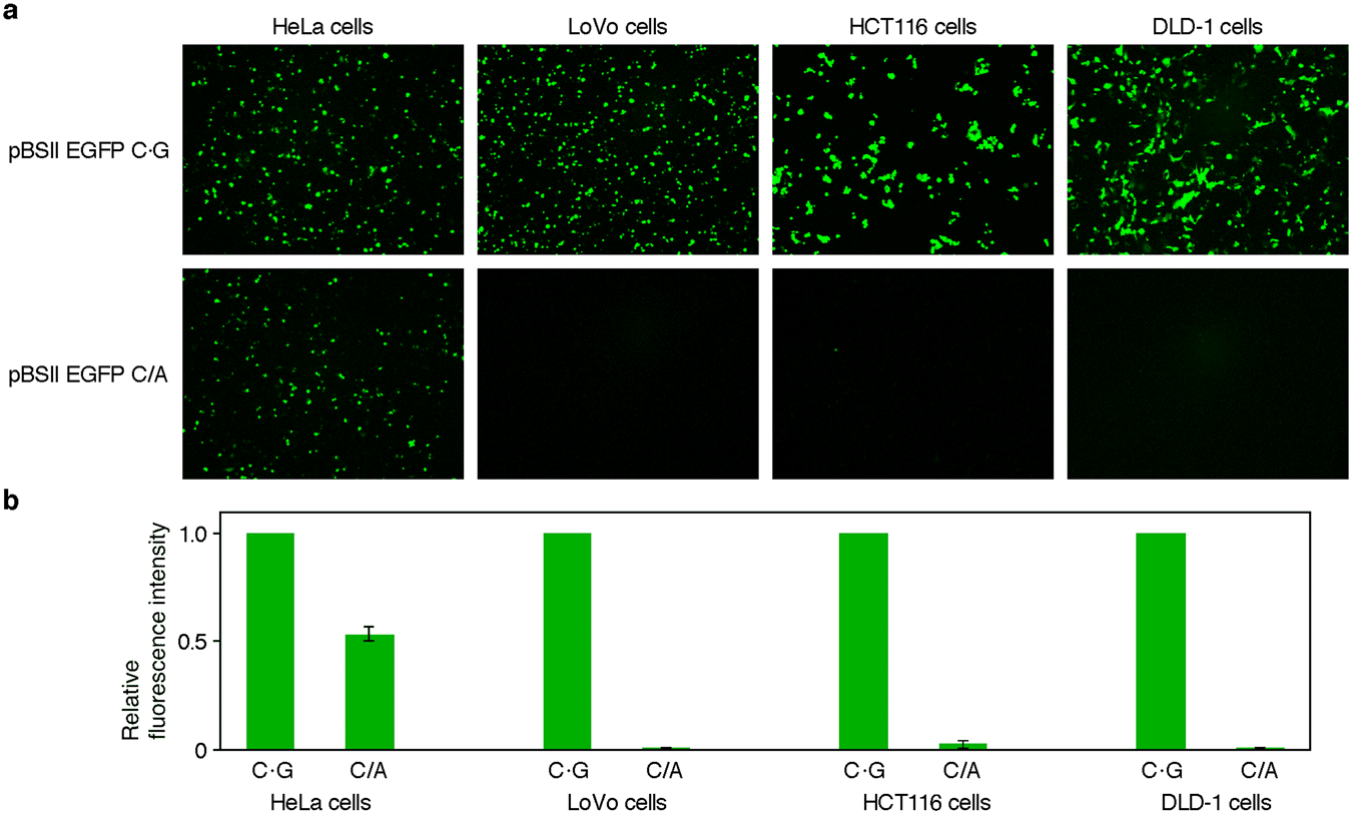
Fluorescence detection of MMR in human cells. (**a**) Fluorescence microscopy images of the cells transformed with the plasmids containing the normal and mismatched EGFP genes. HeLa cells were used as a positive control, and three types of MMR-deficient cells were tested. The cells were transformed with pBSII EGFP C·G and pBSII EGFP C/A, which were designed to express the normal and mismatch-containing EGFP genes, respectively, and observed with a fluorescence microscope after an incubation at 37 °C for 24 h. (**b**) Comparison of the fluorescence observed for the cells transformed with pBSII EGFP C/A to that observed for each positive control (pBSII EGFP C·G). The fluorescence intensities in panel **a** were quantified using the ImageJ software, and the averages of the results obtained for each cell in three independent experiments are shown with error bars, representing standard deviations.

To begin with, HeLa and LoVo cells, which are proficient and deficient in MMR, respectively, were transformed with pBSII EGFP C·G containing the normal EGFP gene, and the production of the fluorescent protein was confirmed by observing the cells with a fluorescence microscope. In these experiments, an overnight culture after the transformation was required to obtain sufficient fluorescence intensities (Supplementary Fig. S1). Next, the cells were transformed with pBSII EGFP C/A, which contained the C/A mismatch in the EGFP gene. As the MMR-deficient cells, we also used HCT116 and DLD-1, which are human colon cancer cell lines similar to the LoVo cells, but derived from different origins^29^. Fluorescence was detected in the HeLa cells transformed with this plasmid (Fig. 2a), and the quantitative analysis of the micrographic images revealed that the fluorescence intensity was about one-half of that observed for the same cells transformed with pBSII EGFP C·G (Fig. 2b). In contrast, fluorescence was hardly visible in all of the MMR-deficient cell types (Fig. 2). The experiments were repeated at least three times, and the results were quite reproducible.

### Confirmation of transformation using the tdTomato gene expression

**Figure 3:**
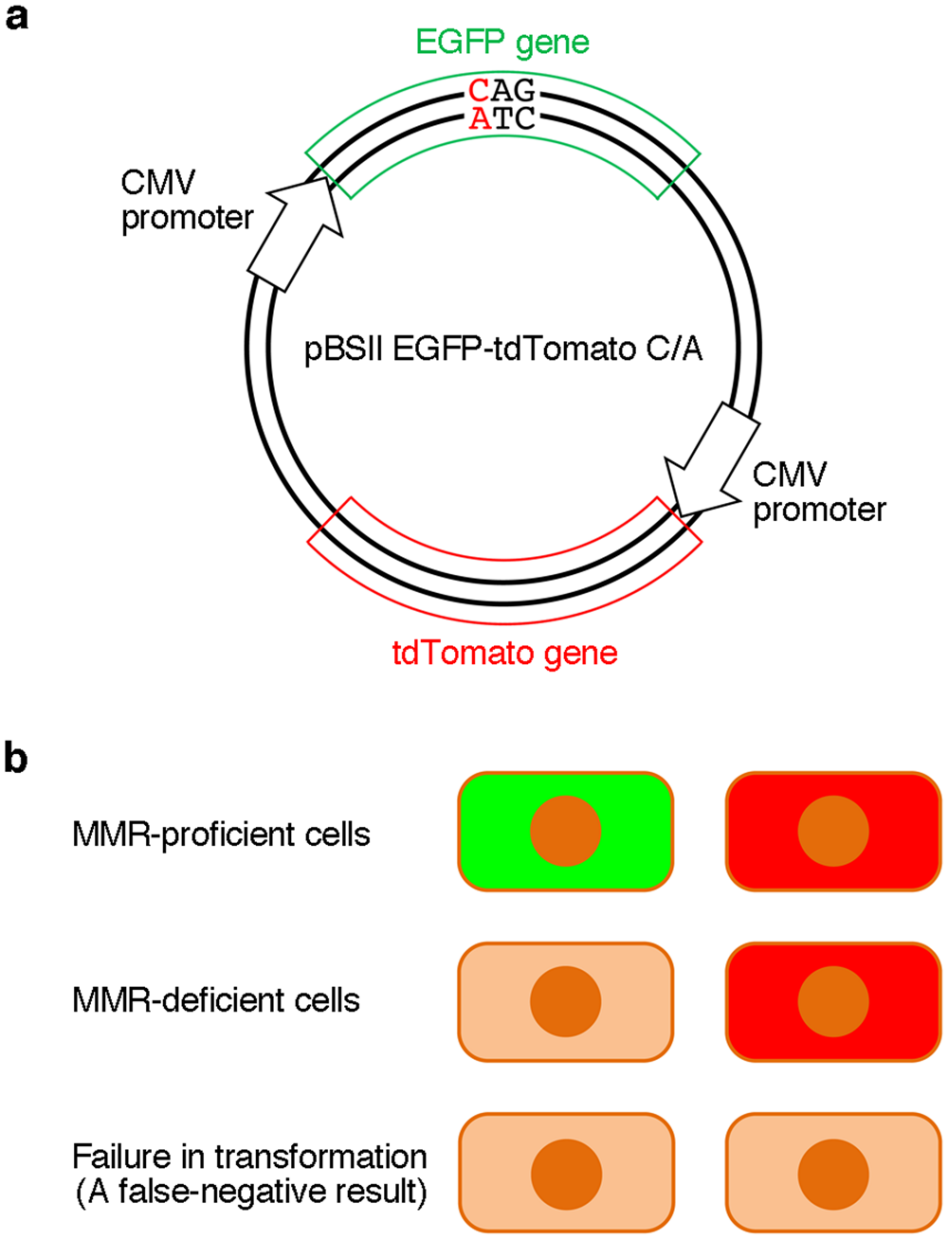
Dual detection to prevent false-negative results. (**a**) The plasmid used in this study. The tdTomato gene to be transcribed from the CMV promoter was inserted into pBSII EGFP, as shown in Fig. 4, and a C/A mismatch was produced in the EGFP gene, in the same manner as in pBSII EGFP C/A. (**b**) Expected results of the experiments using pBSII EGFP-tdTomato C/A. When the cells are transformed with this plasmid, the tdTomato gene will be expressed, and the red fluorescence would be detected, regardless of the cellular MMR ability. The observation of this red signal indicates the successful transformation, and can be used to prevent false-negative results.

**Figure 4:**
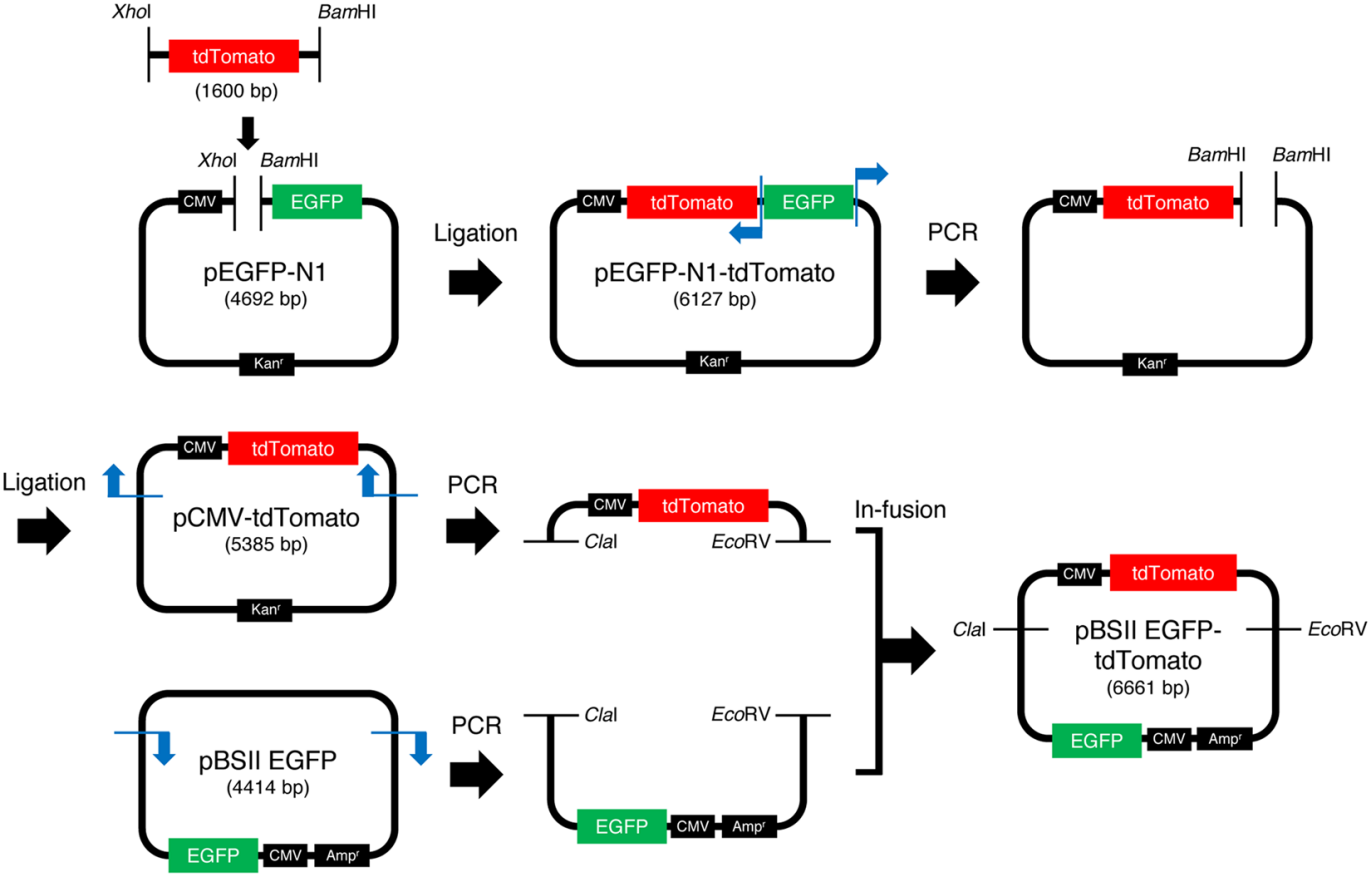
Construction of pBSII EGFP-tdTomato. The tdTomamo gene was obtained by PCR using wtTDP43tdTOMATOHA (Addgene), and blue arrows indicate the PCR primers. The details are described in Methods.

**Figure 5:**
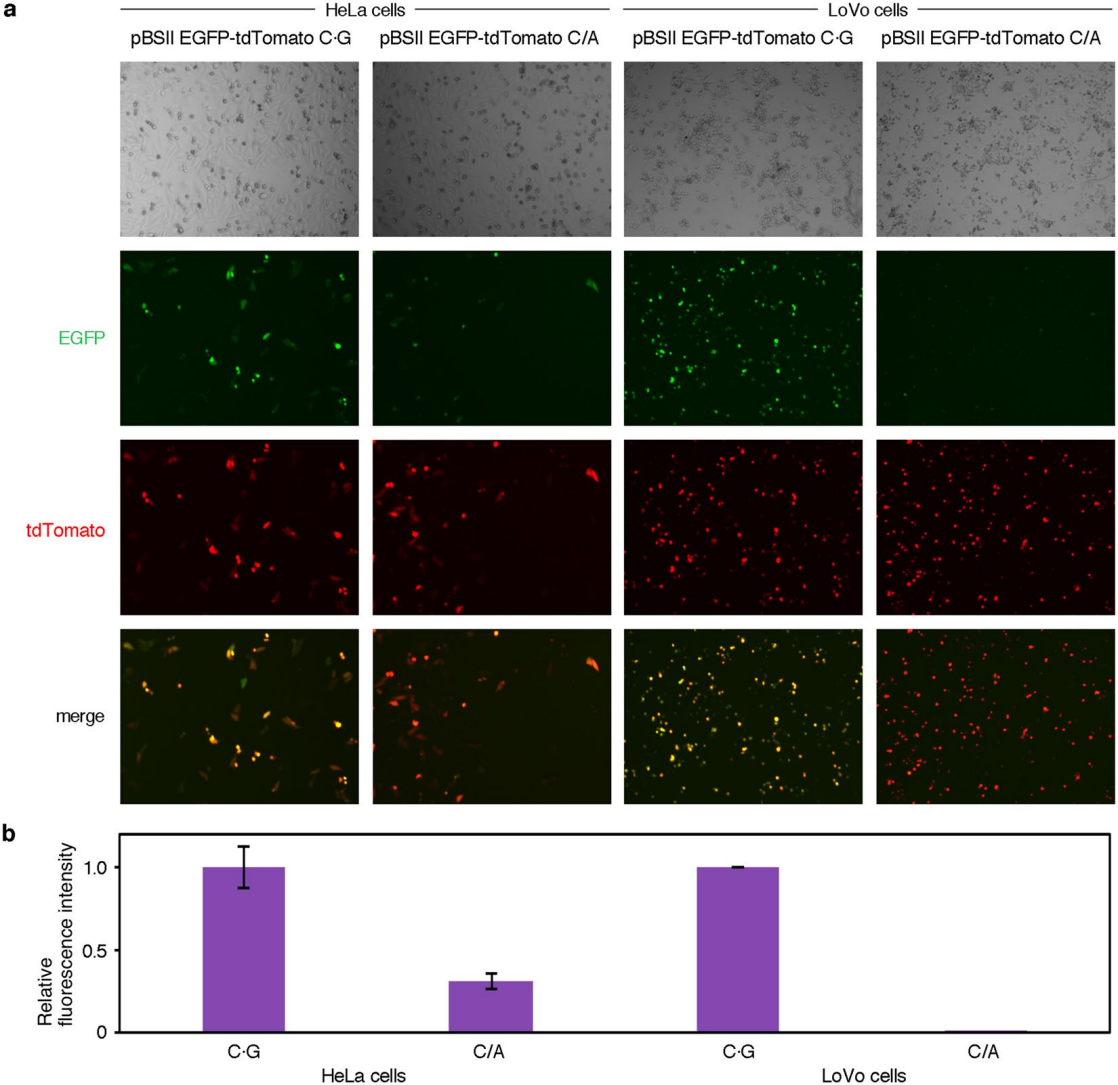
(**a**) Fluorescence microscopy images of the cells transformed with the plasmids containing the tdTomato gene. For the observation of the EGFP gene expression, the excitation and detection wavelengths were 470 and 535 nm, respectively, and the same microscopic fields were observed at 605 nm with excitation at 540 nm to detect tdTomato. HeLa and LoVo cells were used as MMR-proficient and deficient cells, respectively. (**b**) Comparison of the EGFP fluorescence normalized by tdTomato. The fluorescence intensities were quantified using the BZII-analyzer software, and the EGFP/tdTomato ratios were compared between the C·G- and C/A-containing plasmids.

Since fluorescence is detected only when the correct EGFP gene is expressed, there is no possibility of obtaining false-positive results in this method. However, a failure in transformation results in false-negative observations, in which the fluorescence from MMR-proficient cells is not detected. To avoid such errors, we tried to improve the method. A gene encoding a red fluorescent protein, tdTomato^30^, which is transcribed from the CMV promoter, was inserted into the EGFP gene expression plasmid, as shown in Fig. 3a. In practice, pBSII EGFP-tdTomato was prepared by ligating the PCR product containing the CMV promoter and the tdTomato gene with the large fragment of pBSII EGFP (Fig. 4). Subsequently, the C/A mismatch was produced in the EGFP gene in the same manner as in pBSII EGFP C/A. HeLa and LoVo cells were transformed with this plasmid (pBSII EGFP-tdTomato C/A, Fig. 3a) and the counterpart without the mismatch (pBSII EGFP-tdTomato C·G), with the expectations that the red fluorescence of tdTomato would be detected when the transformation is successful, while the green fluorescence of EGFP would indicate the cellular MMR ability (Fig. 3b). After an incubation at 37 °C for 24 h, the transformed cells were observed with a fluorescence microscope at two wavelengths (Fig. 5a). As expected, the red fluorescence was detected regardless of the cellular MMR ability, and the expression of the repaired EGFP gene was observed only in the MMR-proficient HeLa cells. Although there was an obvious difference in the green fluorescence between the HeLa and LoVo cells transformed with pBSII EGFP-tdTomato C/A, the number of the green fluorescent HeLa cells was considerably smaller than that observed for the positive control (pBSII EGFP-tdTomato C·G) (Fig. 5b).

**Figure 6:**
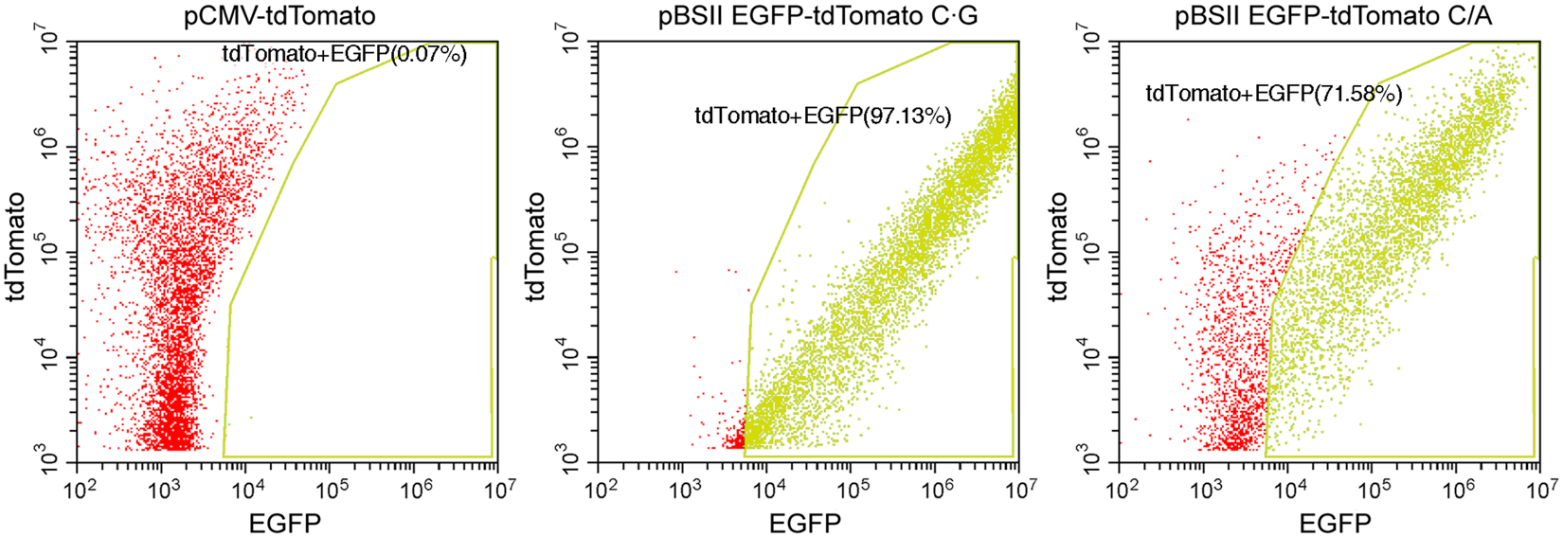
Analysis of the fluorescent HeLa cells by flow cytometry. The results are displayed on logarithmic scales of the fluorescence intensities of EGFP and tdTomato. The red and yellow dots represent the cells expressing only the tdTomato gene and both of the tdTomato and correct EGFP genes, respectively. The percentages of the latter cells are also shown. The full data sets are shown in Supplementary Fig. S2.

To obtain more information, the cells were analyzed by flow cytometry (Fig. 6 and Supplementary Fig. S2). When the HeLa cells were transformed with pBSII EGFP-tdTomato C·G, a significant correlation was observed between the fluorescence intensities of EGFP and tdTomato because the two genes were designed to be coexpressed in each cell. However, in the experiment using pBSII EGFP-tdTomato C/A, about 30% of the cells did not show the EGFP fluorescence, probably due to the mismatch remaining in the EGFP gene, and the fluorescence intensity of EGFP was relatively low.

## Discussion

In this study, we have developed a method for the fluorescence detection of the cellular MMR ability, using the EGFP gene expression system. In the MMR-proficient cells, the normal EGFP gene is generated by the repair of the mismatch, as shown in Fig. 1c, whereas the stop codon in the mRNA transcribed from the mismatch-containing gene inhibits the production of the full-length EGFP, if MMR does not function in the cells. There were two concerns at the beginning of this study. One was whether a plan to introduce a nick to initiate the MMR pathway should be executed, and the other was whether replication, which would produce the normal EGFP gene, would occur in the cultured cells. Judging by the results obtained in this study, neither of these two points needed to be considered. It was assumed that the nick was produced by a cellular nuclease, as expected from our previous study^23^, and the replication was avoided by using the pBlueScript vector, which lacks the eukaryotic replication origin.

This study is the first visualization of *in vivo* MMR, and it is important that the correlation of the fluorescence detection with the mismatch correction was demonstrated experimentally. The method described here may be applied to Lynch syndrome screening. The difference between this method and the MSI and IHC tests, which are currently used for Lynch syndrome screening, is that the entire process of MMR is monitored directly in this method, and there is no possibility of obtaining false-positive results because fluorescence is detected only when MMR is completed. False-negative results can be avoided by using the pBSII EGFP-tdTomato system (Fig. 3). In the experiments using pBSII EGFP-tdTomato C/A, the ratio of the HeLa cells showing the green fluorescence was lower (Fig. 5b) than that observed for the HeLa cells transformed with pBSII EGFP C/A (Fig. 2b), and the reproducibility of the results was confirmed by changing the experimenter. This may be because pBSII EGFP-tdTomato C/A (6661 bp) is longer than pBSII EGFP C/A (4414 bp). Since the plasmid is randomly nicked in the transformed cells, the particular nick at a position appropriate for the initiation of MMR may be formed at a lower frequency when the plasmid size is large. More information was obtained from the results of the flow cytometry analysis (Fig. 6). In the case of the cells transformed with pBSII EGFP-tdTomato C/A, about 30% of the cells were found in the EGFP-negative region (shown in red), and even in the EGFP-positive region, the fluorescence intensity was lower than that observed for the cells transformed with pBSII EGFP-tdTomato C·G. Not only the unrepaired plasmid but also this low intensity is thought to be the reason for the reduced fluorescence intensity in the quantification of the microscopy images (Fig. 5b).

In addition to Lynch syndrome, there are several other aspects where MMR is important in cancer treatment. Recently, immune checkpoint inhibitors have attracted attention in cancer immunotherapy. Activated T cells, which induce apoptosis of the target cancer cells, have an immune checkpoint protein called programmed cell death 1 (PD-1) on their surfaces, and the binding of one of the two ligands for this protein (PD-L1 and PD-L2) expressed on the surfaces of the cancer cells to PD-1 inhibits the cytotoxic activity of the T cells^31^. Therefore, antibodies to PD-1 can maintain the anticancer immune response of the T cells^32^. However, the clinical benefit of an immune checkpoint blockade with pembrolizumab, a monoclonal antibody to PD-1, reportedly depends on the MMR status, and MMR-deficient tumors are more responsive to the PD-1 blockade^33^. Moreover, adjuvant chemotherapy with fluorouracil also depends on the MMR activity. The treatment improved the overall survival among the patients with MSS and MSI-L tumors, but there was no benefit in the MSI-H group^34^.

As described above, the importance of analyzing the MMR status of cells is increasing. The simple fluorescence method developed in this study will contribute to many research fields ranging from biology to clinical studies, and in particular may improve biomarker-driven personalized diagnosis and therapy for cancer patients.

## Methods

### Preparation of pBSII EGFP

The DNA fragment containing the CMV promoter, the EGFP gene, and the SV40 polyadenylation (poly(A)) signal was amplified by PCR, using PrimeSTAR Max DNA polymerase (Takara Bio), the pEGFP-C1 vector (Takara Bio USA), and two primers with the sequences of 5′-d(GGGGCGCGCGGACAAACCACAACTAGAATG)-3′ and 5′-d(CCCGCGCGCTAGTTATTAATAGTAATCAAT)-3′, in which the underlines indicate the *Bss*HII sites. After purification by 1% agarose gel electrophoresis (Supplementary Fig. S3a), the product was treated with *Bss*HII (New England Biolabs, 5 units) in buffer (10 μl) containing 20 mM Tris-acetate (pH 7.9), 50 mM potassium acetate, 10 mM magnesium acetate, and 100 μg/ml BSA at 50 °C for 1 h. pBlueScript II SK (−) (Agilent Technologies, 2.8 μg) was treated with *Bss*HII (5 units) and Antarctic phosphatase (New England Biolabs, 5 units) in the above buffer (30 μl) at 50 °C for 1 h. The *Bss*HII-treated plasmid and the PCR product were precipitated with ethanol, and dissolved in water (25 μl and 10 μl, respectively). Aliquots of these solutions (1 μl and 3 μl, respectively) were mixed, and after the addition of DNA ligation mix (Takara Bio, 4 μl), the mixture was incubated at room temperature for 30 min. *Escherichia coli* DH5α competent cells (Takara Bio, 50 μl) were mixed with the ligation mixture (4 μl) on ice, and cultured on Luria-Bertani (LB) agar plates with ampicillin overnight. Single colonies were isolated and cultured in LB medium with 50 μg/ml ampicillin (LB Amp) (1 ml) for 6 h. The DNA in each colony was purified with the QIAprep Spin Miniprep Kit (Qiagen) and analyzed by gel electrophoresis after *Bss*HII treatment (Supplementary Fig. S3b). The correct insertion of the PCR product into the plasmid was confirmed by the sequence analysis.

### Preparation of pBSII EGFP-tdTomato

The DNA fragment containing the tdTomato gene was amplified by PCR, using PrimeSTAR Max DNA polymerase (Takara Bio), the wtTDP43tdTOMATOHA (Addgene, plasmid #28205), and two primers with the sequences of 5′-d(CCCCTCGAGCCACCATGGTGAGCAAGGGAG)-3′ and 5′-d(GGGGGATCCTACTTGTACAGCTCGTCCATG)-3′, in which the underlines indicate the *Xho*I and *Bam*HI sites, respectively. After purification by 1% agarose gel electrophoresis, the product was treated with *Bam*HI and *Xho*I (New England Biolabs, 10 units) in buffer (10 μl), containing 50 mM Tris-HCl (pH 7.9), 100 mM NaCl, 10 mM MgCl_2_, and 1 mM DTT, at 37 °C for 1 h. The pEGFP-N1 vector (Takara Bio USA) was also treated with *Bam*HI and *Xho*I (10 units) in the above buffer at 37 °C for 1 h. Aliquots of these solutions were mixed, and then incubated with Ligation high (Toyobo). The constructed plasmid, pEGFP-N1-tdTomato, was amplified by PCR, using PrimeSTAR Max DNA polymerase (Takara Bio) and two primers with the sequences of 5′-d(GGGGGATCCTACTTGTACAGCTCGTCCATG)-3′ and 5′-d(CCCGGATCCGACTCTAGATCATAATCAGCC)-3′, in which the underlines indicate the *Bam*HI sites. The product was treated with *Bam*HI (New England Biolabs), and then incubated with Ligation high (Toyobo), to remove the EGFP gene. This plasmid, pCMV-tdTomato, contained the tdTomato gene between the CMV promoter and the SV40 poly(A) signal, which were the same as those used in pBSII EGFP. The tdTomato expression fragment containing the CMV promoter, the tdTomato gene, and the SV40 poly(A) signal was amplified by PCR, using PrimeSTAR Max DNA polymerase (Takara Bio), the pCMV-tdTomato, and two primers with the sequences of 5′-d(AAAGAACATATCGATGCGTTACATAACTTA)-3′ and 5′-d(TTTGCTCACGATATCTAAGATACATTGATG)-3′ (Supplementary Fig. S4a). In parallel, the pBSII EGFP fragment containing the CMV promoter, the EGFP gene, and the SV40 poly(A) signal in pBlueScript II SK (−) was amplified by PCR, using PrimeSTAR Max DNA polymerase (Takara Bio), the pBSII EGFP, and two primers with the sequences of 5′-d(CCCGATATCGTGAGCAAAAGGCCAGCAAAA)-3′ and 5′-d(CCCATCGATATGTTCTTTCCTGCGTTATCC)-3′ (Supplementary Fig. S4b). The two DNA fragments were then purified using the QIAquick Gel Extraction Kit (Qiagen), and ligated using the In-Fusion HD Cloning Kit (Takara Bio). This newly constructed plasmid, pBSII EGFP-tdTomato, was confirmed by *Cla*I and *Eco*RV digestion (Supplementary Fig. S5) and the sequence analysis.

### Preparation of the mismatch-containing plasmids

*E*. *coli* DH5α cells carrying either pBSII EGFP or pBSII EGFP-tdTomato were grown at 37 °C overnight in LB Amp (4 ml), and incubated with 1 × 10^11^ pfu/ml M13KO7 helper phage (New England Biolabs, 400 μl) at room temperature for 30 min. This mixture was incubated in LB Amp (400 ml) containing 20 μg/ml kanamycin at 37 °C overnight, and after the *E*. *coli* cells were pelleted by centrifugation at 9,000 rpm for 15 min, the supernatant was incubated with DNase I (Takara Bio, 50 units) and RNase A (Takara Bio, 0.4 mg) in the presence of 5 mM MgCl_2_, at 37 °C for 1 h. Polyethylene glycol 6000 (25 g) and NaCl (15 g) were dissolved in this solution, and this mixture was kept at 4 °C overnight. The single-stranded DNA was collected by centrifugation at 15,000 rpm for 20 min, purified by phenol extraction, and precipitated with ethanol. This DNA (50 μg) was mixed with 5′-d(TGCCGTTCTTCTACTTGTCGGCCAT)-3′ (for pBSII EGFP C/A and pBSII EGFP-tdTomato C/A) or 5′-d(TGCCGTTCTTCTGCTTGTCGGCCAT)-3′ (for pBSII EGFP C·G and pBSII EGFP-tdTomato C·G) (0.6 nmol), and was 5′-phosphorylated with T4 polynucleotide kinase (Takara Bio), in buffer (200 μl) containing 33 mM Tris-acetate (pH 7.9), 66 mM potassium acetate, 10 mM magnesium acetate, and 0.5 mM DTT, and incubated at 70 °C for 5 min, at 37 °C for 30 min, and then at room temperature for 20 min. Each primer-hybridized DNA was incubated with T4 DNA polymerase (Takara Bio, 100 units) and T4 DNA ligase (Takara Bio, 1,750 units) in the above buffer, in the presence of 0.5 mM dNTP, 0.5 mM ATP, and 0.01% BSA, at 37 °C overnight. A 1.58 g/ml CsCl solution (1.35 ml) and 10 mg/ml ethidium bromide (0.15 ml) were added to the mixture, and the double-stranded DNA was isolated by ultracentrifugation at 90,000 rpm for 25 h (Supplementary Fig. S6). The ethidium bromide was removed by extraction with butanol, and the CsCl was removed using an Amicon Ultra-4 centrifugal filter unit (Merck Millipore). The quantity and the quality of the DNA were measured by using a NanoDrop One spectrophotometer (Thermo Fisher Scientific).

### Transfection of the cells

The cells were grown overnight in DMEM (HeLa S3), Ham’s F-12 (LoVo), McCoy’s 5A (HCT116), or RPMI-1640 (DLD-1) medium supplemented with 10% (HeLa, HCT116, and DLD-1 cells) or 20% (LoVo cells) fetal bovine serum (FBS), 100× Penicillin–Streptomycin solution (Wako Pure Chemical Industries), and Amphotericin B suspension (Wako Pure Chemical Industries), at 37 °C in a humidified 5% CO_2_ incubator. The cells were seeded into the wells of a TF1205M micro slide glass (Matsunami Glass Industries) or into a 96-well plate, grown to approximately 90% confluence, and transfected with the plasmids (300 ng per well) using Lipofectamine 2000 (Thermo Fisher Scientific, 0.5 μl per well), according to the manufacturer’s instructions. After 24 h, the cells were analyzed with the Olympus IX71 fluorescence microscopy system (pBSII EGFP C·G and C/A) or with the Keyence BZ-9000 microscope (pBSII EGFP-tdTomamo C·G and C/A), and the fluorescence intensities were quantified using the ImageJ 1.50i software (National Institutes of Health) or the BZII-analyzer software (Keyence).

### Flow cytometry

HeLa S3 cells were seeded into a 12-well plate, grown to approximately 80% confluence, and transfected with plasmids (2.4 μg per well) using Lipofectamine 2000 (5 μl per well). After 24 h, cells were harvested and resuspended into D-PBS (Nacalai Tesque) plus 2% FBS, and the suspension was passed through a cell strainer. The obtained cell suspensions were analyzed by CytoFLEX S flow cytometer (Beckman Coulter), in which green (excitation at 488 nm) and yellow (excitation at 561 nm) fluorescence channels were calibrated with HeLa cells transformed with pBSII EGFP and pCMV-tdTomato plasmids, respectively. After gating for debris exclusion and doublet discrimination, 10,000 single cells were analyzed. The data were processed with the CytExpert software (Beckman Coulter).

## Electronic supplementary material

Supplementary Information

## Data Availability

The datasets generated during and/or analyzed during the current study are available from the corresponding author on reasonable request.
