# Supplementary material for: Fluorescence detection of DNA mismatch repair in human cells
Source: Sci Rep. 2018 Aug 15;8:12181. doi: 10.1038/s41598-018-30733-x (PMC6093906; doi:10.1038/s41598-018-30733-x)
Supplement: Supplementary file 1 — Supplementary Information [file 41598_2018_30733_MOESM1_ESM.pdf]

Supplementary Information

Fluorescence detection of DNA mismatch repair in human cells

Shunsuke Ito[1], Miyako Shiraishi[1], Kazuki Tsuchihashi[1], Reine Takatsuka[1], Junpei Yamamoto[1], Isao Kuraoka[1,2] & Shigenori Iwai[1]

[1] Division of Chemistry, Graduate School of Engineering Science, Osaka University, 1-3 Machikaneyama, Toyonaka, Osaka 560-8531, Japan

[2] Present address: Department of Chemistry, Faculty of Science, Fukuoka University, 8-19-1 Nanakuma, Jonan-ku, Fukuoka 814-0180, Japan

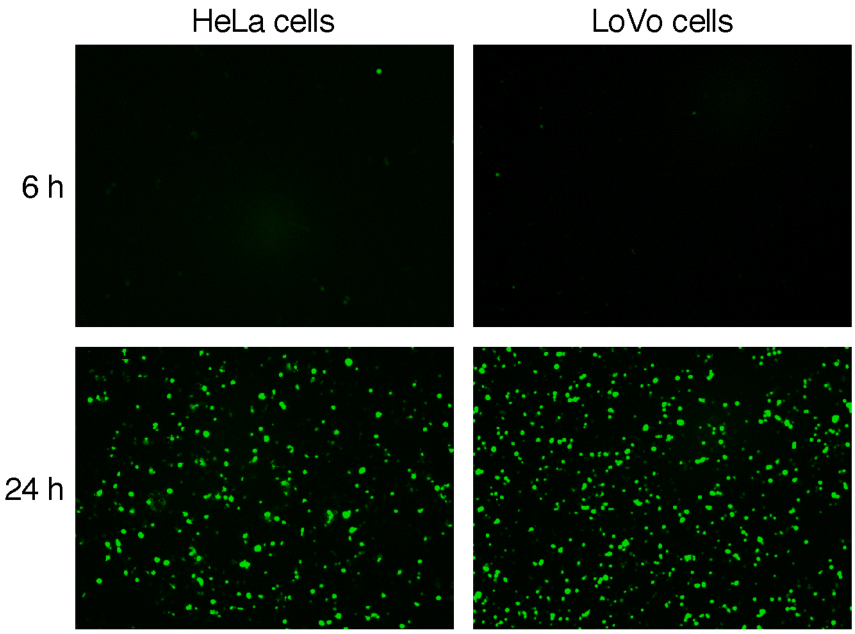

Supplementary Figure S1. Fluorescence microscopy observation of the cells transformed with pBSII EGFP C·G.

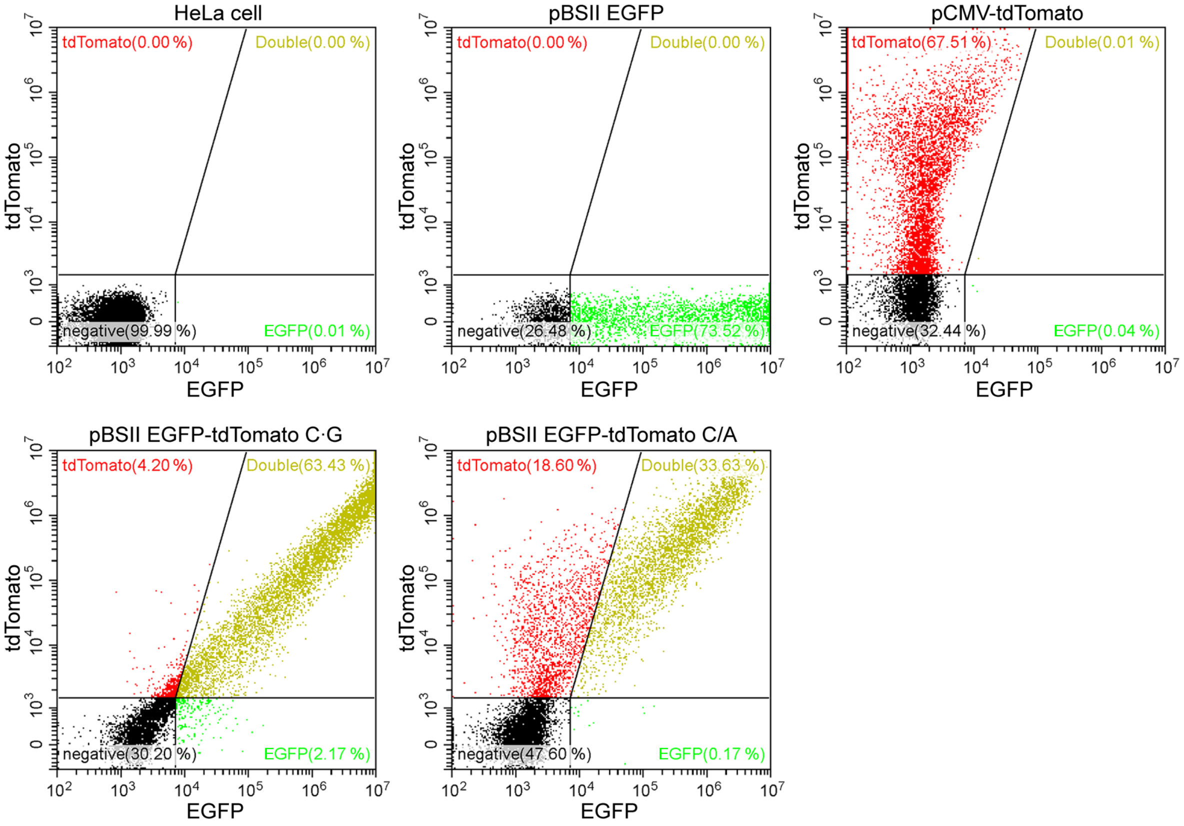

Supplementary Figure S2. Full-scale presentation of the flow cytometry results. The cytograms are divided into four regions, which contain the negative (black), EGFP-positive (green), tdTomato-positive (red), and double positive (yellow) cells.

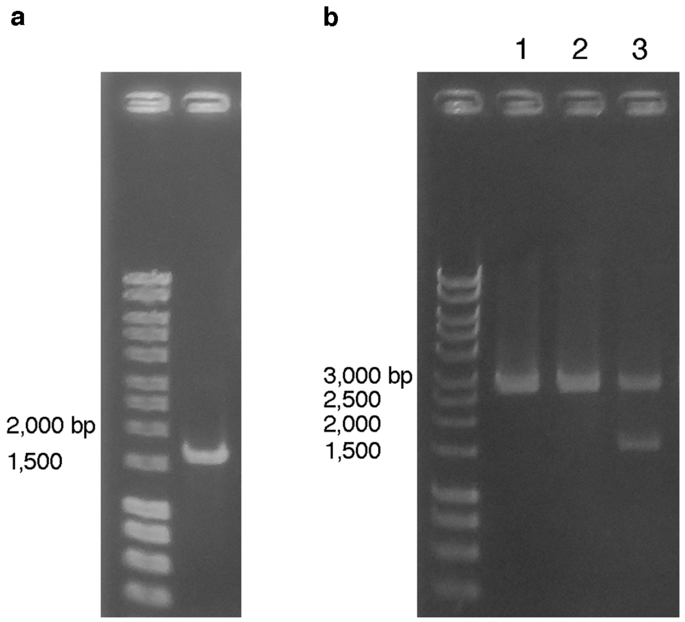

Supplementary Figure S3. (**a**) Analysis of the purified PCR product containing the CMV promoter, the EGFP gene, and the poly(A) signal by 1% agarose gel electrophoresis. (**b**) Analysis of *Bss*HII treatment of the ligation product between pBluescript II SK (-) and the PCR product. Among the plasmids obtained from single colonies #1, #2, and #3 (lanes 1, 2, and 3, respectively), only the plasmid from #3 contained the PCR product shown in panel **a**, and it was confirmed to be pBSII EGFP by the sequence analysis.

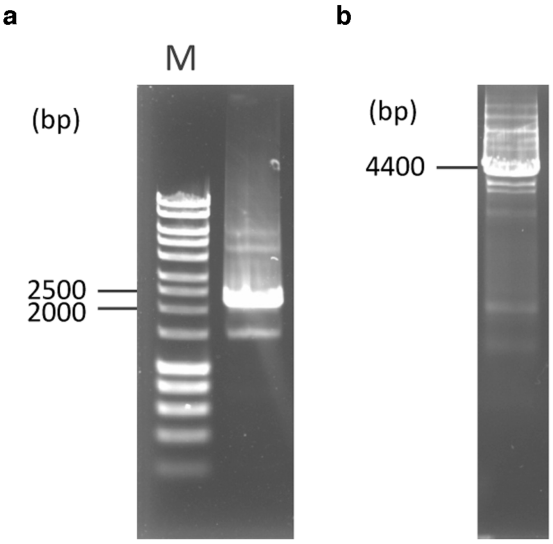

Supplementary Figure S4. Analysis of the PCR products from pCMV-tdTomato (**a**) and pBSII EGFP (**b**). Both of the products had *Cla*I and *Eco*RV sequences at their ends.

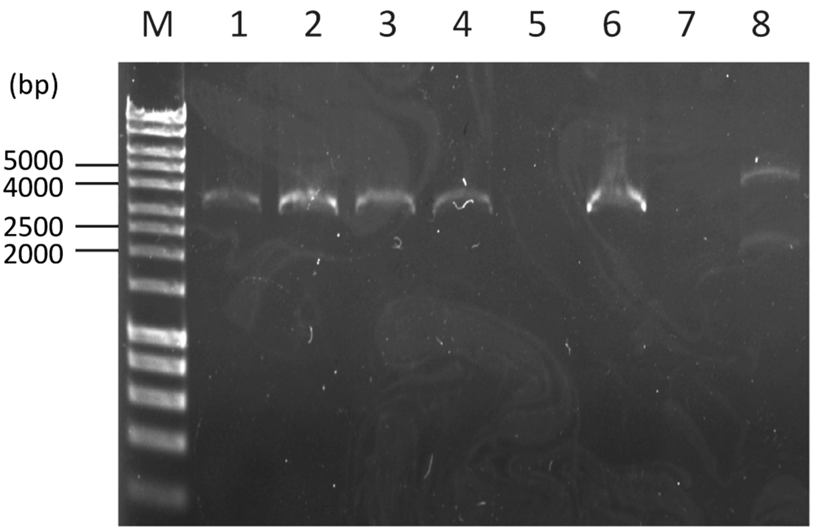

Supplementary Figure S5. Analysis of *Cla*I–*Eco*RV treatment of the fusion products in the preparation of pBSII EGFP-tdTomato. The plasmid in lane 8, which produced two bands with sizes close to 4400 and 2200 bp, was confirmed to be the desired product by the sequence analysis.

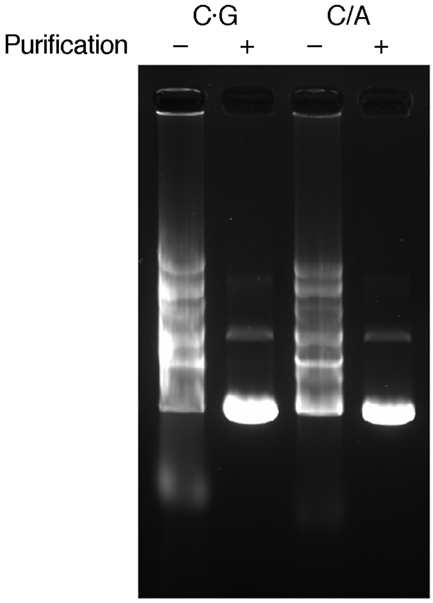

Supplementary Figure S6. Analysis of pBSII EGFP C·G and pBSII EGFP C/A, before and after purification by ultracentrifugation, by 0.8% agarose gel electrophoresis.
